# Deciphering the reproductive protein-protein interaction network in *Anopheles gambiae* with *Drosophila melanogaster* as a framework

**DOI:** 10.1186/gb-2011-12-s1-p32

**Published:** 2011-09-19

**Authors:** Daniel Achinko, Paul Mireji, Flaminia Catteruccia, Dan Masig

**Affiliations:** 1 Molecular Biology and Biotechnology Department, International Center of Insect Physiology and Ecology (icipe), Nairobi 30772-00100, Kenya; 2 Biochemistry and Molecular Biology Department, Egerton University, Njoro, Kenya; 3 Division of Cell and Molecular Biology, Imperial College London, London SW7 2AZ, UK

## Background

Protein-protein interactions (PPIs) are the most fundamental biological processes at the molecular level. The experimental methods for testing PPIs are time-consuming and are limited by analogs for many reactions. As a result, a computational model is necessary to predict PPIs and to explore the consequences of signal alterations in biological pathways. Reproductive control of the vector *Anopheles gambiae* using transgenic techniques poses a serious challenge. To meet this challenge, it would help to define the biological network involving the male accessory gland (MAG) proteins responsible for successful formation of the mating plug [1]. This plug forms in the male and is transferred to the female during mating, hence initiating the PPIs in both sexes. As is the case in *Drosophila melanogaster*, a close relative of *A. gambiae*, some MAG proteins responsible for the formation of the mating plug have been shown to alter the post-mating behavior of females.

## Methods and results

The STRING database for known PPIs was used to identify orthologs of *A. gambiae* proteins in *Drosophila* (Table 1). Twenty-seven proteins are known to form the mating plug in *A. gambiae*, and 16 others were obtained as strings in the STRING database. Chromosome synteny comparisons for proteins with more than 50% identity between species were carried out using the Artemis Comparison Tool (ACT version 9.0), and this showed 24.39% matches (M), 12.20% mismatches (MM) and 63.41% unmatched (NM). The network built in Cytoscape (version 2.8.0) with the UniProt IDs for these *Drosophila* orthologs showed 14 complexes, with 4 of them being for *Drosophila*. The network showed 555 nodes and 2,344 edges. The top 50 identified hubs in the network showed a range of 3 to 30 interactions. The expression values for these proteins in FlyAtlas showed that they are upregulated in the reproductive tissues of both sexes. To understand the processes involved in plug formation, the Reactome database was used, and the hub proteins were identified in 49 of the 2,021 known processes in *Drosophila*. Twelve proteins were involved in the following processes: metabolism of proteins (8.8e–13), gene expression (2.0e–06), 3’-UTR-mediated translational regulation (7.7e–08), regulation of β-cell development (1.3e–06), diabetes pathways (6.8e–06), signal recognition (preprolactin) (5.0e–07) and membrane trafficking (1.3e–03). Of the top 50 proteins, 92% had orthologs in *A. gambiae*, with one identified in the mating plug and four others identified as strings to AGAP009584, which is found in the mating plug. Acp29AB was identified in the network and is known to induce post-mating responses in *Drosophila*, confirming that the network is reproductive and giving an insight into the possible pathways involved. The CG9083 (Q8SX59) protein was ranked first among the hub proteins but has no ortholog in *A. gambiae*. Interestingly, it has the same protein properties as the Plugin protein (AGAP009368) in *A. gambiae*, suggesting that Plugin may be the main protein in the PPI reproductive network in *A. gambiae.* The Whelan and Goldman (WAG) maximum likelihood tree evaluations of the plug proteins in *A. gambiae* and their orthologs in *Drosophila* showed that these proteins are involved in similar biological processes in both species, but the *A. gambiae* protein evaluation provided a better explanation for the expected process as it clustered in both pre-mated and post-mated PPIs.

**Table 1:** Orthologs of *Anopheles gambiae* proteins in *Drosophila* identified using the STRING database

***A. gambiae *** **ID (plug proteins)**	STRING	Chromosome	Sex	**Ortholog in *****Drosophila***	UniProt ID	Chromosome	STRING score	Chromosome synteny
AGAP009099		3R	Male	CG7356	Q9VLU2	2L	108	MM
					Q8IPH0	2L	108	MM
AGAP009368		3R	Male	CG15005	Q9VZG4	3L	40	NM
AGAP009370		3R	Male					
AGAP012830		Unknown	Male			Unknown		
AGAP008276		2R	Male	CG12350	Q7JPN9	3R	139	NM
AGAP008277		2R	Male	CG12350	Q7JPN9		137	MM
AGAP013150 (AGAP004671)			Male	CG4738	Q9VKJ3		362	NM
AGAP005791		2L	Male	CG32834	Q9WIW6	2R	74.7	NM
					D3DMG3			
AGAP007041		2L	Male	CG6676	Q95SM8	2R	172	NM
AGAP006418		2L	Male	CG32679	Q8IRL3	X	166	NM
					D9PTU6			
AGAP009673		3R	Male	CG5976	Q7KTY3	3L	317	MM
					Q0GT94			
AGAP003083		2R	Male	CG6113	Q9VKT9	2L	180	NM
AGAP001649		2R	Male	CG31414	Q8IMY3	3R	537	M
				CG3647	Q4V4A3			
					Q4V4J1			
	AGAP0012412	3L		CG6437	Q9W297	2R	583	NM
	AGAP002055	2R		CG3132	Q9VGE7	3R	642	NM
AGAP009584		3R	Both	CG31884	Q9V429	2L	134	NM
	AGAP000565	X		CG2151	P91938	X	687	M
				CG11401	Q9VNT5	3L	687	
	AGAP011107	3L		CG6852	B7Z076	3L	144	NM
					Q9VVT6	3L	144	
	AGAP010517	3L		SOD1	B8YNX4	3L	302	NM
	AGAP007201	2L		TRX-2	Q6HI1	2L	169	NM
	AGAP007827	3R		CG17654	P15007	2L	736	M
	AGAP009623	3R		CG8893	P07487	X	544	M
	AGAP007120	2L		CG2210	P08879	3R	292	NM
	AGAP006818	2L		CG8975	P48592	2R	588	NM
				CG17797	O46197	2L		
	AGAP010198	3R		CG5371	P48591	2L	1311	M
	AGAP001325	2R		CG32920	Q960M4	3R	238	NM
				CG7217	Q6XHE3	3R		
AGAP012407		3L	Both	CG6988	P54399	3L	642	M
	AGAP007393	2L		CG8983	Q3YMU0	2R	696	NM
	AGAP002816	2R		CG1333	Q9V3A6	3L	582	M
AGAP011630		3L	Female	CG33998	Q6IG52	2R	66	NM
					B3DN29	2R	66	
AGAP004533		2R	Both	CG10992	Q9VY87	X	464	M
AGAP005194		2L	Female	CG5255	Q9VEM5	3R	154	NM
AGAP005195		2L	Female	CG4053	Q9VEM7	3R	136	NM
					Q9VIT3	2L		
					Q8IGA0	2L		
AGAP006904		2L	Female	CG4859	Q9W122	2R	762	M
				CG4859	Q8MLN6	2R		
	AGAP003319	2R		CG6281	Q9VH14	3R	148	MM
AGAP007347		2L	Female	CG7798	A1ZAB8	2R	131	NM
AGAP003139		2R	Both	CG18525	Q9VFC2	3R	293	NM
				CG18525	Q9VFC2	3R		
AGAP006964		2L	Female	CG32147	Q8SZB7	3L	155	NM
	AGAP009172	3R		CG5355	Q9VKW5	2L	980	M
AGAP006420		2L	Both	CG32679	Q8IRL3	X	137	NM
AGAP009212		3R	Both	CG7219	A4V9T5	2L	206	NM
NOVEL ACP1 (from female)		3R: b/w 9370 & 9371	Male					
NOVEL ZCP7 (AGAP008071)		3R: b/w 5051000 & 5067900	Male	CG8564	Q9VS63	3L	164	NM

This table shows the 27 proteins known to be in the mating plug of *A. gambiae*[*1*], derived predominantly from the male. The 16 strings predicted as orthologs in *Drosophila*, using the STRING database, have varying scores. Scores above 60 can be trusted following their alignments. Plugin, which has the lowest score, has no good ortholog in *Drosophila*. Most of the proteins are encoded on chromosome arms 2L and 3R in both species. The chromosome synteny comparisons using ACT showed 24.39% matches (M), 12.20% mismatches (MM) and 63.41% unmatched (NM). The presence of gaps between the alignments resulted in the observed MM and NM. The nucleotide sequences at the chromosomal locations where the proteins NOVEL ACP1 and NOVEL ZCP7 are encoded were used to identify similar proteins and their orthologs.

## Conclusions

The identification of *A. gambiae* proteins in this network creates more targets for functional analysis and reproductive control of the malaria vector.
